# Robustness Evaluation of a Legacy N-Glycan Profiling Method for a Therapeutic Antibody Under ICH Q14 Lifecycle Principles

**DOI:** 10.3390/antib15010009

**Published:** 2026-01-15

**Authors:** Ming-Ching Hsieh, Chao Richard Li, Margaret A. Velardo, Jingming Zhang, Babita S. Parekh

**Affiliations:** 1 Analytical Sciences, Eli Lilly and the Company, Branchburg, NJ 08876, USA; 2 Manufacturing Statistics, Eli Lilly and Company, Indianapolis, IN 46221, USA; 3 Quality Assurance, Eli Lilly and the Company, Branchburg, NJ 08876, USA

**Keywords:** N-glycan, glycosylation, peptide-N-glycosidase F (PNGase F), solid phase extraction (SPE), reagent age, robustness, analytical method lifecycle management, ICH Q14

## Abstract

Background: This study assesses the robustness of a legacy N-glycan profiling method for the therapeutic antibody MAB1 with different Peptide-N-glycosidase F (PNGase F) enzyme sources, solid phase extraction (SPE) cartridges, and reagent stability, aligning with ICH Q14 lifecycle management principles. Glycosylation profiling is critical for therapeutic antibodies as it influences both function and pharmacokinetics. Method: The legacy N-glycan profiling method, 2-aminobenzoic acid (2-AA) labeling combined with normal-phase HPLC, was re-evaluated to confirm consistent analytical performance in the context of evolving regulatory expectations. The evaluation focused on three key factors: PNGase F enzyme sources, solid-phase extraction (SPE) cartridges, and reagent stability. Results: Commercial PNGase F enzymes showed various performances, with some sources yielding significant differences. Several SPE cartridges were also tested, with certain formats displaying poor recovery and high variability, particularly for sialylated glycans. In addition, reagent stability studies revealed rapid degradation of the labeling reagent within a few days. Conclusions: These results underscore the importance of risk control, continual improvement, and lifecycle management to ensure reliable glycosylation analysis and the sustained robustness of legacy methods.

## 1. Introduction

The glycosylation profile is an important characteristic of therapeutic antibodies and other glycoproteins. Using appropriate analytical methodologies allows comprehensive elucidation of glycan structures, which can affect antigen binding, protein folding, stability, effector functions, immunogenicity, pharmacokinetics, and pharmacodynamics [1,2,3]. These analyses play a role in product characterization, quality control, and compliance with regulatory standards. Several analytical methodologies exist for studying glycosylation at the level of intact proteins, glycopeptides, or released glycans. Techniques, such as capillary electrophoresis (CE), mass spectrometry (MS), and high-performance liquid chromatography (HPLC)-based separation methods, including reversed-phase (RP), normal-phase (NP), and hydrophilic interaction chromatography (HILIC), are commonly used [4,5]. Analysis of released glycans can yield detailed structural information and allows for quantitation, in comparison to analyses performed on intact proteins or glycopeptides.

Since released glycans lack an intrinsic chromophore or fluorophore that can be detected by HPLC, it is common practice to chemically label a fluorophore to these glycans via reductive amination [6]. Reductive amination provides a stoichiometric label, resulting in a one-to-one molar ratio of fluorescence tag to glycan. A variety of fluorophores are employed for chemical labeling, such as 2-aminobenzamide (2-AB), 2-aminobenzoic acid (2-AA or anthranilic acid), RapiFlour-MS, InstantAB, 8-aminonaphthalene-1,3,6-trisulfonate (ANTS), and procainamide, selected based on the requirements of analytical detection [5]. The general workflow for fluorescence-labeled N-glycan analysis by HPLC includes: (1) Peptide-N-glycosidase F (PNGase F) digestion; (2) chemical labeling; (3) glycan enrichment using solid phase extraction (SPE); and (4) separation and quantification with a fluorescent detector [6].

A legacy analytical method was established for evaluating the glycosylation profile of the therapeutic antibody MAB1. This approach utilized 2-AA labeling in combination with normal-phase HPLC separation. The choice of 2-AA labeling and normal-phase separation offered several distinct advantages [7]. Notably, 2-AA exhibits nearly double the quantum yield compared to 2-AB, resulting in enhanced sensitivity. Moreover, one-step labeling can be accomplished in salts (acetate-borate)-buffered methanol without substantial desialylation. Additionally, glycans labeled with 2-AA can be effectively separated according to size, linkage, and charge. The developed and qualified method became an integral component within the broader analytical toolbox for MAB1 characterization.

The biopharmaceutical analysis landscape has undergone a significant transformation, and the Q14 concept highlights the importance of systematic method lifecycle management [8]. This approach offers a comprehensive framework to maintain method robustness, accuracy, and consistent performance throughout its application. The legacy glycosylation profile method for MAB1 was developed using conventional approaches with limited flexibility. This methodology is influenced by variables like PNGase F source, SPE materials, and reagent age, which can impact data quality and reproducibility.

To address these challenges, we conducted a systematic evaluation of the impact of enzyme source, SPE material, and reagent aging on the performance of the glycosylation profiling method for MAB1. This assessment follows the principles outlined in ICH Q14, which call for a structured approach to managing the entire lifecycle of an analytical method. By proactively identifying and controlling sources of variability, such as enzyme and SPE source differences and reagent stability, we aim to mitigate risks that could compromise data reproducibility. This systematic strategy ensures that the method remains robust and reliable throughout its intended lifecycle, supporting consistent analytical outcomes and regulatory compliance. Furthermore, the study emphasizes the importance of continuous verification and adaptability, enabling the method to maintain fit-for-purpose under the operational conditions.

## 2. Experimental

### 2.1. Materials

The IgG_1_ therapeutic antibody (MAB1) used in this study was produced by Eli Lilly and Company, with a 2 mg/mL Reference Standard applied in all experiments. PNGase F enzymes from various brands were sourced from New England Biolabs (Ipswich, MA, USA for P0704L, P0705L, P0705S), Agilent Technologies (Santa Clara, CA, USA for AdvanceBio GKE-5005B), Promega (Madison, WI, USA for V4831), Thermo Fisher Scientific (Waltham, MA, USA for GIBCO A39245), and N-Zyme Scientifics (Doylestown, PA, USA for NZPP050 via Bulldog Bio Inc.).

Different solid phase extraction (SPE) cartridges were purchased from the following companies: Waters Corporation (Milford, MA, USA) for OASIS HLB, OASIS PRiME HLB, and GlycoWorks HILIC, Supelco/Millipore Sigma (Bellefonte, PA, USA) for ENVI-Carb^TM^ and Discovery^®^ DPA-6S, and Thermo Fisher Scientific (Waltham, MA, USA) for Hypercarb^TM^ HyperSep^TM^.

Chemicals were obtained from various sources. Anthranilic acid or 2-aminobenzic acid (2-AA), 2-methylpyridine borane complex, acetic acid, ammonium hydroxide, β-mercaptoethanol, boric acid, sodium acetate trihydrate, sodium dodecyl sulfate, tetrahydrofuran, and triethylamine were from Sigma-Aldrich (St. Louis, MO, USA). Acetonitrile was obtained from Fisher Chemical (Waltham, MA, USA). Methanol was obtained from J.T. Backer (Avantor, Radnor, PA, USA). Detergent NP-40 was from New England Biolabs (Ipswich, MA, USA).

### 2.2. N-Linked Glycan Release and Labeling

In each sample preparation, approximately 80 µg of MAB1 was denatured and reduced by 60 µL of 0.5% sodium dodecyl sulfate (SDS) and 1% β-mercaptoethanol in the presence of 0.25–0.3% ammonium hydroxide for 5 min at pH ~9.5, 100 °C. Once the mixture reached ambient temperature, 20 µL of 5% NP-40 (in water) and 2 µL of PNGase F enzyme were added to the reaction vial. The solution was then incubated at 37 °C overnight (~18 h). The released glycans were labeled with anthranilic acid via reductive amination, using 2-methylpyridine borane complex as the reducing agent at 80 °C for 90 min. Specifically, 10 µL of the labeling solution containing 30 mg/mL anthranilic acid and 20 mg/mL 2-methylpyridine borane complex in 4% (*w*/*v*) sodium acetate-trihydrate and 2% (*w*/*v*) boric acid in methanol was used for labeling. The PNGase F from New England Biolabs (P0705L) is used in the legacy glycosylation method for MAB1.

### 2.3. Enrichment of Labeled N-Linked Glycan Using Solid Phase Extraction (SPE) Cartridges

The labeled glycans were enriched through solid-phase extraction cartridges by gravity, using 95% acetonitrile (1 mL × 4) to wash away the excess reagents and 0.5 mL of 20% acetonitrile to elute the labeled glycans. The 2-AA-labeled glycans were subsequently injected into a normal-phase HPLC. The SPE cartridge, OASIS from Waters, is used in the legacy glycosylation method for MAB1.

The samples were generally prepared independently in accordance with the established procedures, which were described in Section 2.2. For SPE evaluation, 2-AA-labeled glycans were pooled before being distributed uniformly among the various SPE cartridges to ensure baseline consistency.

### 2.4. Normal-Phase HPLC

The 2-AA-labeled glycans were separated by a normal-phase HPLC equipped with a fluorescent detector (FLD) at Excitation 315 nm, Emission 400 nm, using Asahipak-NH2P-50 4D, 4.6 × 150 mm 100 Å, 5 µm (Resonac America, Inc., San Jose, CA, USA). Solvent A contains 2% acetic acid, 1% tetrahydrofuran in acetonitrile, and Solvent B is made of 5% acetic acid, 1% tetrahydrofuran, and 3% triethylamine in water. The gradient begins with 30% B for 2 min, followed by a linear increase to 80% B over 68 min. Subsequently, solvent B is increased to 95% in 0.1 min and maintained for an additional 4.9 min. The column temperature is maintained at 50 °C.

### 2.5. Statistical Methods for Data Analysis

Analysis of variance (ANOVA) was performed using JMP^®^ Pro (Version 17.2.0) to evaluate alternative enzymes and SPE cartridges in comparison with the control sources, specifically New England Biolabs’ P0705L enzyme and the OASIS SPE cartridge. A one-way ANOVA was performed, followed by Dunnett’s test, to compare the mean of each alternative group against the mean of the control group.

To assess trends in percent Peak Area for seven-selected peaks over time, each response was analyzed using linear regression with respect to the ages of denaturation solution, enzyme, labeling solution, and labeling reagent, also using JMP^®^ Pro (Version 17.2.0).

## 3. Results

### 3.1. N-Linked Oligosaccharide Profiling of MAB1 by Normal-Phase HPLC

**Figure 1:**
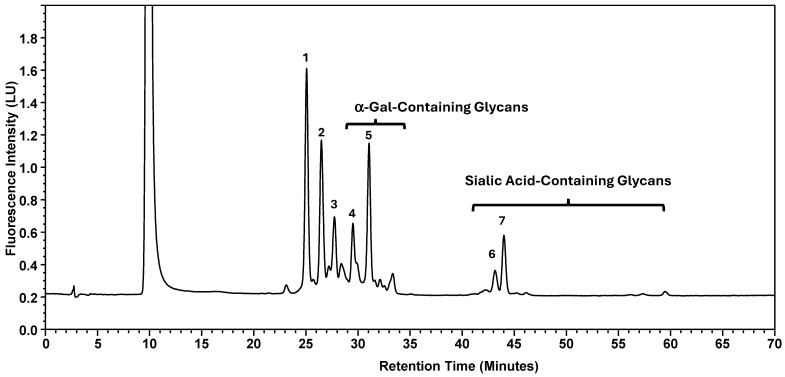
Normal-Phase Chromatography of N-Linked Oligosaccharide Profiling of MAB1. Seven-selected peaks are labeled. The identities of the respective peaks are summarized in Table 1. LU: Luminescence Unit.

Normal-phase chromatography effectively separates both neutral and sialylated glycans, with the sialylated glycans being eluted later due to their hydrophilicity and size, as illustrated in Figure 1, using the nominal PNGase enzyme and SPE cartridges (i.e., New England Biolabs P0705L and OASIS). Seven major glycan peaks were chosen for robustness evaluations (Table 1); while these peaks may not be fully pure, primary components are identified. Peaks 1–5 represent neutral glycans: G0F, G1F, and G2F for Peaks 1–3, respectively, and Peaks 4–5 contain galactose-α-1,3-galactose linkage (α-Gal) glycans. The sialic acid, or N-glycolylneuraminic acid (NGNA)-containing glycans appear in Peaks 6–7. Both α-Gal- and NGNA-containing glycans are critical quality attributes (CQAs) for MAB1 due to the potential immunogenicity.

### 3.2. Evaluation of Enzyme PNGase F from Different Vendors/Packages

PNGase F is commonly used in laboratories to examine N-linked glycosylation in glycoproteins [9]. Multiple manufacturers offer this enzyme in various packages within the US market. In this study, the enzyme sources and packages listed in Table 2 were selected to evaluate the robustness of the enzyme under the fixed experimental protocol described in Experimental 2.2. All these PNGase F enzymes were recombinant from *Elizabethkingia miricola* (formerly *Flavobacterium meningosepticum*) and expressed in *Escherichia coli* (*E. coli*), except the one from GIBCO, for which the source is not identified.

**Figure 2:**
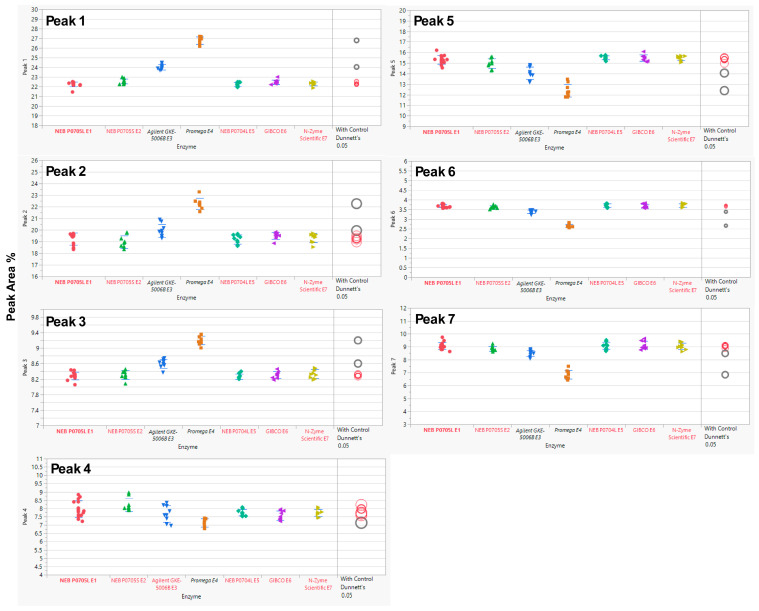
Dunnett’s Comparison of PNGase F Between the Control Enzyme (New England Biolabs P0705L, E1) and Other Enzymes (E2–E7). Means for each enzyme were compared to the control (E1) using ANOVA with Dunnett’s test. Statistically significant differences from the control enzyme (E1, bold red) are shown in black (enzyme labels and circles). Red indicates no significant difference from the control. The circle size reflects the standard deviation of measurements. The *y*-axis represents the % Peak Area, which is the relative abundance of each peak in relation to the total integrated peaks from 20 to 65 min.

Each MAB1 was analyzed in 3 independent assays with three replicates each, except E1 (P0705L), which served as the nominal control and was assessed in 5 independent assays with three replicates. The experiments were performed over three or five independent assays to ensure comprehensive evaluation of both intra-assay and inter-assay analytical variability. In this evaluation, the SPE cartridge OASIS (the nominal cartridge) was used throughout the glycan preparations. Analysis of variance (ANOVA) was performed using JMP^®^ Pro (V17.2.0) to determine the equivalency of enzymes from alternative sources relative to the control enzyme (New England Biolabs’ P0705L or E1). ANOVA with a control group (Dunnett’s test) was utilized to compare the means of each enzyme with the control (E1). The results indicated that two enzyme sources, Agilent’s GKE-5006B and Promega’s V4831, exhibited statistically significant differences from the control enzyme E1 (Figure 2) across all seven selected peaks, with the exception of Peak 4, for which only Promega’s enzyme was significantly different. A summary of the overall *p*-values for each enzyme source alongside their performance on the seven selected peaks is provided in Table 3.

### 3.3. Evaluation of the Solid Phase Extraction (SPE) Cartridges

Solid phase extraction (SPE) cartridges were employed to concentrate fluorescently labeled N-linked glycans and clean up excess reagents. Several SPE cartridge types, as indicated in Table 4, were assessed using a standardized protocol with 95% acetonitrile for adsorption and 20% acetonitrile for elution. The evaluation of robustness involved examining the consistency of glycan recovery across different cartridges using the same elution conditions. PNGase F from New England Biolabs (P0705L, or E1) was used to release the glycans. To reduce procedural variability, a sufficient amount of labeled glycans (greater than 21 mL) from each preparation was pooled and equally distributed, 1 mL to each cartridge, with each type of SPE undergoing triplicate in an assay. A minimum of four to five assays were conducted with triplicate per cartridge, while the OASIS control cartridge was tested in nine separate assays.

**Figure 3:**
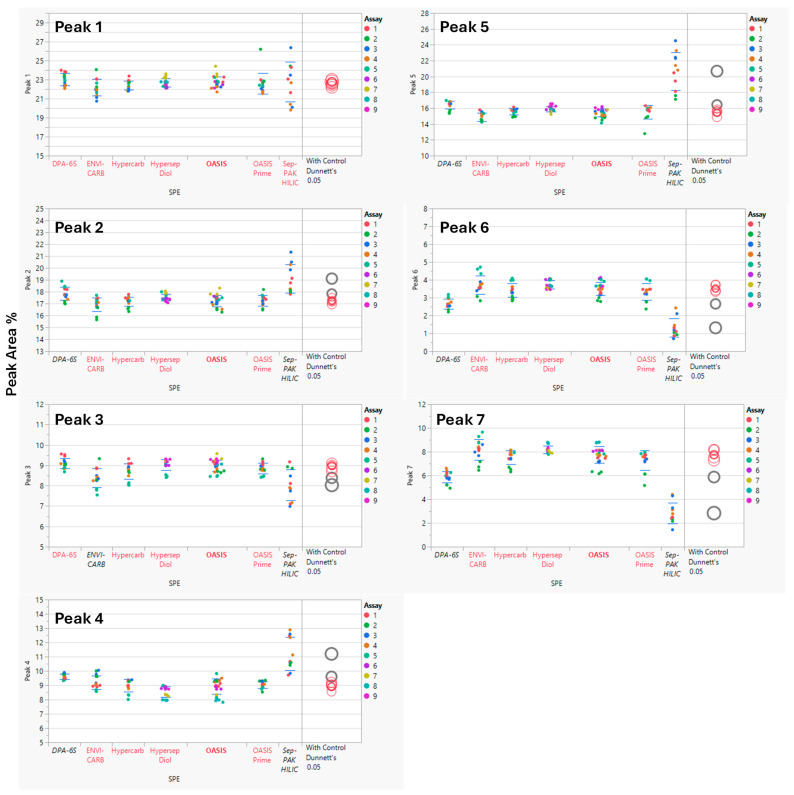
Comparison of Solid Phase Extraction (SPE) Cartridge Performance Using Dunnett’s Test. The control group consisted of the OASIS SPE cartridge, while the comparison groups included the DPA-6S, ENVI-CARB, Hypercarb, HyperSep Diol, OASIS PRiME, and Sep-PAK HILIC cartridges. Statistically significant differences from the control SPE (OASIS, bold red) are shown in black (enzyme labels and circles). Red indicates no significant difference from the control. The circle size reflects the standard deviation of measurements. The *y*-axis represents the % Peak Area, which is the relative abundance of each peak in relation to the total integrated peaks from 20 to 65 min.

Analysis of Variance (ANOVA) was performed using statistical software JMP^®^ Pro (V17.2.0) to compare SPE cartridge results. The Sep-PAK HILIC cartridge showed higher variability and lower recovery of sialic acid-containing glycans (Peaks 6 and 7) than other cartridges, indicating retention or elution issues. ANOVA with a control group (Dunnett’s test) indicated statistically significant differences in several glycan peaks for Sep-PAK HILIC and DPA-6S when compared to the control cartridge, OASIS. Additionally, the ENVI-CARB cartridge demonstrated a minor variation in Peak 3 relative to the control (OASIS), while other SPE cartridges, including HyperSep DIOL, Hypercarb, and OASIS PRiME, produced results comparable to the control (Figure 3). A summary of the overall *p*-values for each SPE cartridge and their performance on the seven selected peaks is provided in Table 5.

### 3.4. Evaluation of the Age of Reagents

**Figure 4:**
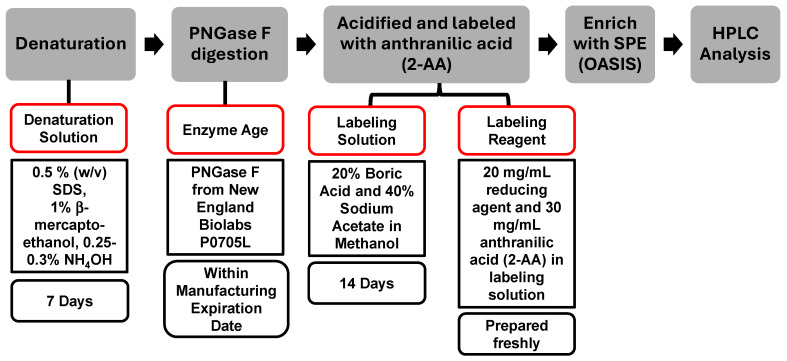
The Sample Preparation Scheme and the Standard Expiration Date of Reagents Used in the Standardized Procedure. The categories outlined in red boxes indicate the specific areas assessed in this study.

The reagents selected for an assay play a crucial role in achieving a consistent result. Expiration dates for reagents are commonly determined based on conservative practices without detailed scientific assessment. In this study, the denaturation solution, enzyme age, labeling solution, and labeling reagent were evaluated, while PNGase F from New England Biolabs (P0705L) and OASIS SPE cartridge were used throughout the assessment. The compositions of these reagents and standard expiration dates are summarized in Figure 4.

**Figure 5:**
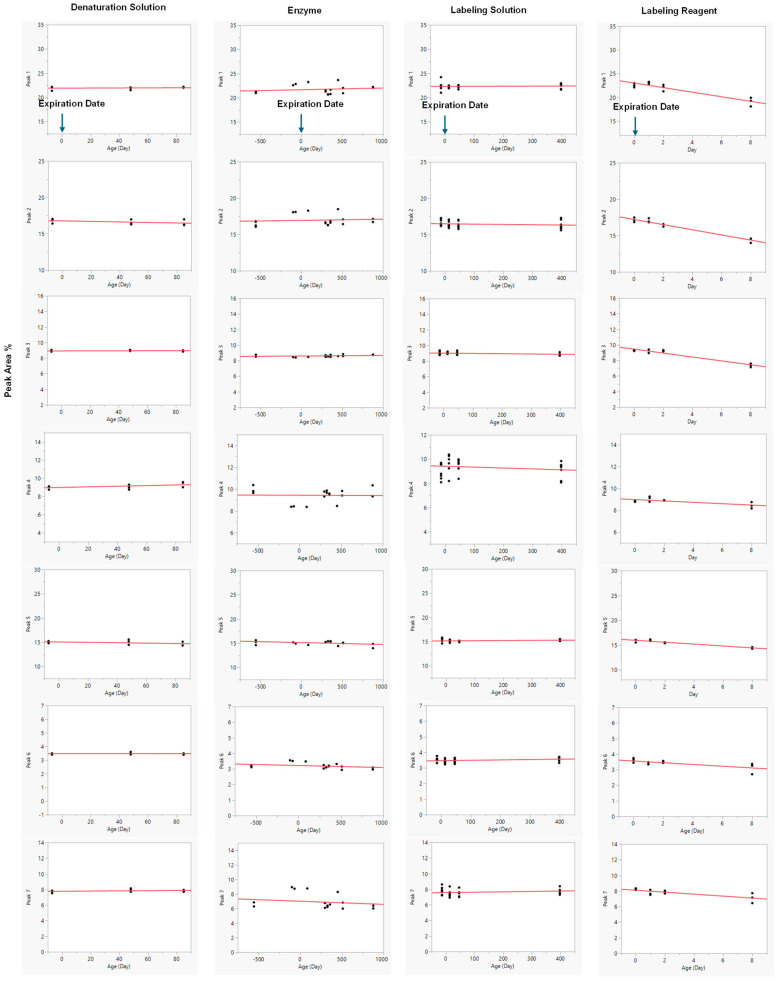
The Linear Regression Model Fit for the Different Ages of Reagents. Day 0 represents the original expiration date. Data were obtained either before the original expiration date (to the left of Day 0) or after the original expiration date (to the right of Day 0).

Reagents of different ages were evaluated for robustness, as illustrated in Figure 5. In each assessment, one variable was either within or beyond its expiration date, while all other reagents were maintained within their respective expiration periods. For example, when investigating the impact of denaturation buffer age, samples tested included those at −7 (prior to expiration), 48-, and 85-day post-expiration; all other reagents, such as the enzyme, labeling solution, and labeling reagent, remained within their stated expiration dates.

The overall data clearly showed that the denaturation solution, enzyme, and labeling solution maintained consistent analytical performance for up to 85, 877, and 398 days past expiration dates, respectively. The denaturation and labeling solutions were stored at ambient temperature, while the PNGase F enzyme (NEB P0705L) was maintained at 2–8 °C. These reagents demonstrated substantial stability, ensuring consistent results over extended periods of time.

Linear regression model fit was performed for each response across varying ages of denaturation solution, enzyme, labeling solution, and labeling reagent (Figure 5). Linear regression analysis revealed that the anthranilic acid and 2-methylpyridine borane complex labeling reagents exhibited reduced stability, as evidenced by a decrease in peak area percentage over time. Conversely, the ages of the denaturation solution, enzyme (NEB P0705L), and labeling solution exerted minimal influence on the analytical results, as evidenced by negligible, near-zero regression slopes. The corresponding *p*-values for these slopes were all greater than 0.05, indicating that they were not statistically different from zero and confirming the absence of significant degradation or change over the duration of the study. In accordance with ICH Q1E principles, these reagents (denaturation buffer, enzyme, and labeling solution) were therefore considered stable, as no significant time-dependent changes were observed in the percent peak areas of the seven-selected peaks. As a result, the slope-related error term was excluded from the prediction interval, as it did not contribute to variability in the stability assessment. The near-zero slopes and their minimal associated error terms indicate a high degree of precision and confidence in the calculated values, further supporting the conclusion of reagent stability. The mean measurements for the denaturation solution, enzyme, and labeling solution exhibited relative standard deviations (RSDs) of less than 5% throughout the study, supporting the analytical accuracy and consistency of the method. In contrast, the labeling reagent showed clear instability over time, as indicated by a high coefficient of determination (R^2^) and highly significant *p*-values (< 0.05). A summary of all analytical parameters is provided in Table 6.

**Figure 6:**
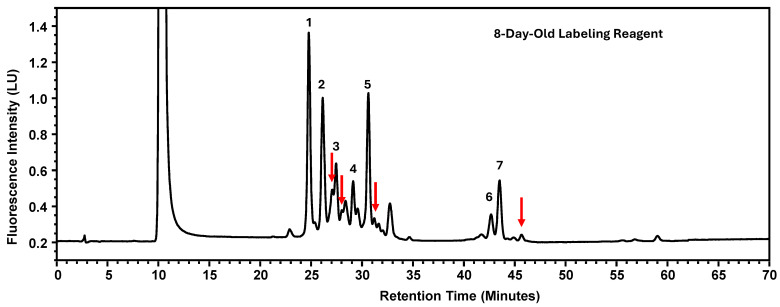
The Glycosylation Profile of MAB1 Using Eight-Day-Old Labeling Reagents. The additional peaks are indicated as red arrows. LU: Luminescence Unit.

Instability of the labeling reagents was associated with reduced labeling efficiency, which would typically result in lower signal intensity for labeled glycans while maintaining similar relative peak area percentages. However, contrary to this expectation, a decrease in percent peak areas was observed. Further investigation revealed that chromatograms generated using labeling reagents aged for eight days exhibited additional, non-target peaks (Figure 6). The presence of these extraneous peaks redistributed the overall peak area, thereby reducing the relative percent peak areas of the seven designated glycan peaks. It should be noted that the reagents had been kept at ambient temperature without protection from light. Drawing from the experimental findings, the newly recommended expiration dates for these four parameters are consolidated in Table 7.

**Table 6:** The Summary of the Parameters of the Linear Model Fits for Denaturation Solution, Enzyme, Labeling Solution, and Labeling Reagent.

Reagent	Response 7-Selected Peaks	Linear Regression Model Statistics				
		R^2^	Slope	*p*-Value (Slope)	Intercept	*N*
Denaturation Solution	Peak 1	0.02	0.0009 ± 0.0002	0.72	21.9 ± 0.1	10
	Peak 2	0.2	−0.004 ± 0.003	0.21	16.8 ± 0.1	10
	Peak 3	0.02	0.0003 ± 0.0008	0.72	8.93 ± 0.04	10
	Peak 4	0.3	0.003 ± 0.002	0.14	9.0 ± 0.1	10
	Peak 5	0.2	−0.004 ± 0.003	0.24	15.1 ± 0.2	10
	Peak 6	0.003	−0.00009 ± 0.0006	0.87	3.48 ± 0.03	10
	Peak 7	0.1	0.001 ± 0.001	0.37	7.78 ± 0.07	10
Enzyme	Peak 1	0.03	0.0004 ± 0.0005	0.47	21.7 ± 0.2	18
	Peak 2	0.01	0.0002 ± 0.0004	0.69	16.9 ± 0.2	18
	Peak 3	0.06	0.00007 ± 0.00007	0.33	8.60 ± 0.03	18
	Peak 4	0.0003	0.00002 ± 0.0003	0.94	9.4 ± 0.2	18
	Peak 5	0.2	−0.0004 ± 0.0002	0.09	15.1 ± 0.1	18
	Peak 6	0.1	−0.0001 ± 0.00009	0.16	3.22 ± 0.04	18
	Peak 7	0.04	−0.0004 ± 0.0005	0.43	7.0 ± 0.3	18
Labeling Solution	Peak 1	0.004	0.0002 ± 0.0006	0.74	22.3 ± 0.1	29
	Peak 2	0.02	−0.0004 ± 0.0005	0.47	16.5 ± 0.1	29
	Peak 3	0.1	−0.0004 ± 0.0002	0.09	9.01 ± 0.04	29
	Peak 4	0.03	−0.0007 ± 0.0008	0.37	9.4 ± 0.2	29
	Peak 5	0.05	0.0003 ± 0.0003	0.26	15.15 ± 0.06	29
	Peak 6	0.08	0.0002 ± 0.0002	0.14	3.47 ± 0.03	29
	Peak 7	0.03	0.0005 ± 0.0005	0.34	7.6 ± 0.1	29
Labeling Reagent	Peak 1	0.85	−0.48 ± 0.06	<0.0001	23.0 ± 0.3	12
	Peak 2	0.94	−0.36 ± 0.03	<0.0001	17.2 ± 0.1	12
	Peak 3	0.92	−0.25 ± 0.02	<0.0001	9.4 ± 0.1	12
	Peak 4	0.51	−0.06 ± 0.02	0.009	8.98 ± 0.08	12
	Peak 5	0.90	−0.19 ± 0.02	<0.0001	15.94 ± 0.08	12
	Peak 6	0.50	−0.06 ± 0.02	0.01	3.56 ± 0.07	12
	Peak 7	0.58	−0.13 ± 0.03	0.004	8.1 ± 0.1	12

**Table 7:** The Newly Recommended Expiration Date for the Four Variables.

Variables	Denaturation Solution	PNGase F Enzyme (NEB P0705L)	Labeling Solution	Labeling Reagent
Original Expiration Date	7 Days	Manufacturing expiration date	14 days	Prepare freshly
Recommended Expiration Date	3 Months	A year beyond the manufacturing expiration date	At least 6 Months	Prepare freshly

## 4. Discussion

Managing the lifecycle of the glycosylation profile analytical method for MAB1, in alignment with ICH Q14, requires ensuring robustness, accuracy, and consistent performance throughout its application [16]. Applying the “fit for purpose” principle, multiple aspects of the methodology were assessed for potential risks and evaluated to determine the robustness of sample preparation. The analytical target profile (ATP) and control strategy for this legacy method are unchanged; however, the sources and packaging of enzymes, types of SPE, and shelf-life of critical reagents have been systematically reviewed.

The glycosylation profile of MAB1 was demonstrated using normal-phase chromatography with 2-AA labeling (Figure 1). Seven representative peaks were selected to evaluate the robustness of different sources/packages of the enzyme, solid phase extraction, and reagent ages.

The objective of the study was strictly limited to a comparative performance assessment of three specific independent variables: the source of PNGase F enzymes, the vendor of solid-phase extraction (SPE) cartridges, and the age of reaction solutions. The analysis was conducted by comparing each treatment group to a single, fixed control condition (the original method) using one-way ANOVA followed by Dunnett’s test, an established procedure specifically designed to compare several different groups to a single control, while ensuring that the family-wise error rate remained controlled. It is also important to note that this investigation did not involve predefined acceptance criteria, performance specifications, or quality thresholds for the seven peak-area percentages. The intent was exclusively comparative, aiming to determine whether variations in enzyme sources, SPE vendors, or reagent ages yielded responses that were statistically distinct from those observed with the original method. Because no equivalence margins or practical significance thresholds were defined, equivalence testing and assessments of practical significance are not applicable in this analysis.

### 4.1. Robustness Evaluation of Enzyme PNGase F from Different Vendors/Packages

Among the tested PNGase F, Agilent GKE 5006B and Promega V4831, exhibited statistically significant differences for the seven selected peaks, as presented in Figure 2. These results may be associated with enzyme concentrations, as both sources may contain lower enzyme concentrations (Table 2). The reported concentration of PNGase F from Agilent (≥2.5 U/mL) is considerably lower than that provided by New England Biolabs (500,000 U/mL). The observed discrepancy likely arises from differences in the definition of enzyme units. Agilent defines one unit as the quantity of enzyme required to catalyze the release of N-linked oligosaccharides from 1 µmol of denatured ribonuclease B per minute at pH 7.5 and 37 °C [17]. In contrast, New England Biolabs (NEB) specifies one unit as the amount of enzyme necessary to remove over 95% of carbohydrate from 10 µg of denatured RNase B within one hour at 37 °C in a 10 µL reaction volume [18]. When PNGase F from Agilent was evaluated using NEB’s assay conditions, it was determined that Agilent’s enzyme exhibited an activity equivalent to 150,000 U/mL [15]. The observed difference does not imply that PNGase F from Agilent or Promega is ineffective; instead, their performance can vary depending on specific conditions, such as when the denaturing buffer has a higher pH. Under such conditions, enzymes with higher concentrations may be able to cleave the complex sialic acid-containing glycans (Peaks 6 and 7) more efficiently. Under the given analytical procedures, only the enzymes from New England Biolabs (P0705S, P0704L), GIBCO (A39245), and N-Zyme Scientifics (NZPP050) showed equivalency to New England Biolabs P0705L, irrespective of the presence of EDTA or glycerol in the formulation buffers, making them suitable substitutes for P0705L.

### 4.2. Robustness Evaluation of the Solid Phase Extraction (SPE) Cartridges

Solid phase extraction (SPE) is a widely adopted technique for sample preparation [19,20], with a broad selection of commercially available cartridges that operate via different mechanisms. In this study, several SPE cartridges with varied modes of action (refer to Table 4) were selected to assess the robustness of the SPE protocol. The methodology involved loading samples containing 95% acetonitrile, subsequently washing with 95% acetonitrile to eliminate excess reactants, and finally eluting with 20% acetonitrile.

Among the seven solid-phase extraction cartridges evaluated, Sep-PAK HILIC and Discovery DPA-6S differed significantly from the OASIS cartridge. HyperSep DIOL, Hypercarb, and OASIS PRiME performed similarly to OASIS, while ENVI-CARB showed a difference only for Peak 3 (Figure 3).

The OASIS cartridge has been used since the method’s development approximately twenty years ago. It consists of a copolymer of divinylbenzene (lipophilic) and vinylpyrrolidone, creating a hydrophilic-lipophilic balance (HLB) sorbent that is suitable for low molecular weight polar compounds [21]. The OASIS PRiME cartridge is a newer version that does not require conditioning and removes interfering matrix components more efficiently [22]. Experimental data show no statistical differences between these similar cartridges.

HyperSep DIOL has short alkyl chains on the surface with polar hydroxyl groups, and therefore, it is suitable for normal-phase retention via hydrogen bonding and dipole–dipole interactions [23]. The data also showed the similarity between HyperSep DIOL and OASIS cartridges.

On the other hand, both ENVI-CARB and Hypercarb cartridges are made of graphitized carbon with non-porous and porous particles, respectively. Both structures are flat sheets of hexagonally arranged carbon atoms with strong π-π interactions. Porous graphitized carbon (PGC) chromatography has been applied to separate isomeric glycans in conjunction with mass spectrometry [24,25] or for glycan enrichment [26], while ENVI-CARB is primarily utilized as a SPE cartridge for sample cleanup [24,27]. A statistical difference was observed between ENVI-CARB and the OASIS cartridge for Peak 3, and the standard deviations were higher compared to other cartridges, except for Sep-PAK HILIC. One possible explanation is that ENVI-CARB retains analytes strongly and requires strong elution conditions, such as the addition of trifluoroacetic acid [24].

The Discovery^®^ DPA-6S cartridge has been designed for the extraction of phenolic compounds as well as other neutral or weakly polar molecules. The sorbent is a polyamide resin, and the primary binding mechanism is reversed-phase retention through hydrogen binding [28]. DPA-6S has been used for cleaning up the anthranilic acid-labeled oligosaccharides from glycosphingolipid [29] or maltoheptaose [30]. The statistical difference between DPA-6S and the control OASIS cartridge may result from negatively charged sialic acid-containing glycans (Peaks 6 and 7), which may not interact strongly with the polyamide resin due to the lack of electrostatic or hydrophilic interactions.

Observations indicated that the Sep-PAK HILIC cartridge resulted in reduced recovery of overall glycans (Peaks 1–7), as judged by the peak heights in chromatograms, and exhibited considerable variability in terms of percent peak areas (Figure 3). This outcome is likely attributable to the legacy elution protocol employing 20% acetonitrile. For improved recovery, particularly with sialic acid-containing glycans, a gentler basic aqueous solution, such as 100 mM ammonium acetate in 5% acetonitrile, may be applied to efficiently elute highly retained glycans from the HILIC cartridge [31]. Nonetheless, Sep-PAK HILIC cartridge is not suitable for this legacy method using standardized procedures.

It is important to accurately assess the amount of sialic acid-containing glycans in MAB1 due to the potential immunogenicity. The MAB1 antibody, manufactured from a mouse cell line, contains sialic acid in the form of N-glycolylneuraminic acid (NGNA), known to be immunogenic in humans [32]. A review of FDA-approved antibodies revealed that 16 were produced using the NS0 cell line and 8 with the Sp2/0 cell line [33]. The immunogenic properties of NGNA may contribute to the accelerated clearance of therapeutic antibodies via anti-NGNA antibody-mediated mechanisms, potentially reducing the circulating half-life and overall effectiveness [32,34]. Given the potential immunogenicity associated with sialic acid-containing glycans in MAB1, accurate and reliable quantification of the NGNA CQA is essential. It is recommended that SPE cartridges that underestimate NGNA levels be excluded from the analytical methodology. This is crucial for both the robustness of an analytical method and for a comprehensive risk assessment related to MAB1’s safety and pharmacokinetic profile.

### 4.3. Robustness Evaluation of the Age of Reagents

A linear regression model was applied to assess a continuous variable and examine changes over time. Results showed that reportable values (% Peak Area) remained stable with the denaturation solution for about three months, the labeling solution for over a year, and the enzyme for more than two years. In contrast, the labeling reagents, particularly anthranilic acid, degraded within eight days, reducing the percent peak areas for all seven selected peaks. The reduction in the selected peak percentage was attributed to the appearance of additional, unidentified peaks (Figure 6). While the specific identities of these new peaks remain undetermined, they may represent the side-products of anthranilic acid degradation, such as aniline formed via decarboxylation during photodegradation. Aniline can be incorporated into glycans via reductive amination with less efficiency [35]. Anthranilic acid is highly reactive and can form various derivatives [36]. In the presence of boric acid, sodium acetate, and methanol, it may undergo esterification to produce boronate esters or methyl anthranilate. Additionally, it can be oxidized to generate quinonoid derivatives. The exact identities of these non-targeted peaks are deemed worthwhile exploring in the future. As such, it is advisable to prepare the labeling reagent freshly, following the current procedure.

### 4.4. Following the Principles of ICH Q14

ICH Q14 emphasizes analytical procedure lifecycle management, where changes are evaluated based on their impact on method performance and product quality. Two major categories are being considered: the objective of the analytical glycosylation method and the performance criteria that the method must meet [37]. The legacy analytical method is used to assess the glycosylation patterns of MAB1 and quantify specific N-linked glycans (% peak area). Acceptance criteria for these glycans were established based on overall manufacturing consistency and analytical variability. The present study evaluated changes in enzyme sources, SPE cartridges, and reagent ages under the method’s Established Conditions (ECs), as defined in the ATP and supported by previous robustness data. No modifications were made to the analytical procedure itself; its intended use remains unchanged, and the method continues to satisfy established performance criteria. Results showed no effect on reportable values for five out of seven enzyme sources and four out of seven SPE cartridges. If these enzyme sources and SPE cartridges are incorporated into current procedures as alternatives, revalidation of this analytical method may not be required.

Furthermore, knowledge management plays a critical role in systematically capturing, evaluating, and curating information throughout the method’s lifecycle. Documentation pertaining to the use of enzyme sources, SPE cartridges, and the shelf-life of reagents is governed by established principles of enzyme digestion, SPE binding and elution mechanisms, as well as the understanding of reagent stability effects on performance. This documentation is maintained through internal change control procedures or comparability study reports. When evaluating enzyme sources, SPE cartridges, and reagent shelf-life, several risks were identified. These include variability and efficiency of enzymes, recovery and selectivity associated with SPE cartridges, and the potential for degraded reagents to compromise accuracy or precision. The risk control was demonstrated in this study through systematic assessments using the pre-established acceptance criteria, ensuring the changes do not introduce unacceptable risk to the method performance.

## 5. Conclusions

The robustness study demonstrates that the analytical method for N-linked oligosaccharide profiling is reliable and stable when evaluated under ICH Q14 principles. The results confirm that changes in enzyme sources (specifically PNGase F from New England Biolabs, GIBCO, and N-zyme Scientifics), as well as the use of different SPE cartridges (OASIS PRiME, HyperSep DIOL, and Hypercarb), do not significantly impact the accuracy or consistency of the method. Additionally, the age of most solutions does not notably affect the results, except for the labeling reagent, which degrades quickly and requires fresh preparation to maintain the best performance.

## Figures and Tables

**Table 1:** The Structures of the Seven Selected N-Linked Glycans from MAB1.

Peak Number	N-Linked Glycan Name	N-Linked Glycan Structure
1	A2G0F	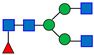
2	A2G1F	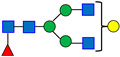
3	A2G2F	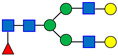
4	A2G3F	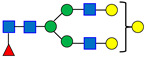
5	A2G4F	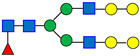
6	A2G2FS1	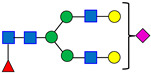
7	A2G3FS1	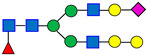

A: antennae, G: galactose, F: fucose, S: sialic acid. Monosaccharide residues are N-acetylglucosamine (GlcNAc, 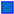), fucose (Fuc, 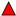), mannose (Man, 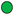), galactose (Gal, 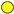), and N-glycolylneuraminic acid (NGNA, 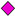).

**Table 2:** The Properties of the PNGase F from Different Enzyme Sources and Packages.

Enzyme Symbol	Enzyme Source	Catalog Number	Assay	Replicate # in Each Assay	Concentration (U/µL)	Formulation
E1	New England Biolabs	P0705L [10]	5	3	500	20 mM Tris-HCl, 50 mM NaCl, 5 mM EDTA, pH 7.5 at 25 °C
E2	New England Biolabs	P0705S [10]	3	3	500	20 mM Tris-HCl, 50 mM NaCl, 5 mM EDTA, pH 7.5 at 25 °C
E3	Agilent Technologies	AdvanceBio GKE-5006B [11]	3	3	≥0.0025 ^a^	20 mM Tris-HCl, 50 mM NaCl, 1 mM EDTA, pH 7.5
E4	Promega Corporation	V4831 [12]	3	3	10	20 mM Tris-HCl, 50 mM NaCl, 5 mM EDTA, pH 7.5 at 25 °C
E5	New England Biolabs	P0704L [10]	3	3	500	20 mM Tris-HCl, 50 mM NaCl, 5 mM EDTA, 50% Glycerol, pH 7.5 at 25 °C
E6	GIBCO	A39245 [13]	3	3	500	Not Available
E7	N-Zyme Scientifics	NZPP050 [14]	3	3	500	PBS (137 mM NaCl, 10 mM phosphate, 2.7 mM KCl, pH 7.4

#: number. ^a^ The concentration is equivalent to 150 U/µL based on the enzyme unit used by New England Biolabs [15].

**Table 3:** Statistical Summary of ANOVA Results for Enzyme Comparison.

Parameter	*p*-Value						
	NEB P0705L E1	NEB P0705S E2	Agilent GKE-5006B E3	Promega E4	NEB P0704L E5	GIBCO E6	N-Zyme Scientifics E7
Peak 1	1.0000	0.0542	<0.0001	<0.0001	1.0000	0.2310	0.9997
Peak 2	1.0000	0.5972	0.0024	<0.0001	1.0000	0.5801	0.9979
Peak 3	1.0000	0.9865	<0.0001	<0.0001	0.9953	0.9943	0.7780
Peak 4	1.0000	0.4444	0.3030	<0.0001	0.6013	0.0835	0.5313
Peak 5	1.0000	0.2345	<0.0001	<0.0001	0.8115	0.8979	0.9735
Peak 6	1.0000	0.6011	<0.0001	<0.0001	0.8984	1.0000	0.9482
Peak 7	1.0000	0.3447	<0.0001	<0.0001	1.0000	0.9945	1.0000

**Table 4:** Solid Phase Extraction (SPE) Cartridges Used in This Study.

Cartridge	Solid Phase	Type	Manufacturer	Bed Weights	Volume	Assay #	Replicate # in Each Assay
OASIS	Copolymer of divinylbenzene and N-vinylpyrrolidone	Polymeric reversed phase	Waters	10 mg	1 mL	9	3
OASIS PRiME	Copolymer of divinylbenzene and N-vinylpyrrolidone	Polymeric reversed phase	Waters	30 mg	1 mL	5	3
DPA-6S	Polyamide	Reversed phase	Supelco Sigma-Aldrich	50 mg	1 mL	5	3
HyperSep DIOL	Diol-functionalized silica	Normal phase	Fisher Scientific	50 mg	1 mL	5	3
Hypercarb	Porous Graphitic Carbon (PGC)	Graphitized carbon	Fisher Scientific	25 mg	1 mL	5	3
ENVI-Carb	Graphitized Non-Porous Carbon (GNP)	Graphitized carbon	Supelco Sigma-Aldrich	100 mg	1 mL	5	3
Sep-PAK HILIC	Silica-based aminopropyl sorbent	Hydrophilic Interaction	Waters	10 mg	1 mL	4	3

#: number.

**Table 5:** Summary of ANOVA Results for SPE Cartridge Comparison.

Parameter	*p*-Value						
	DPA-6S	ENVI-CARB	Hypercarb	HyperSep Diol	OASIS	OASIS Prime	Sep-PAK HILIC
Peak 1	0.8767	0.2283	0.7603	0.9993	1.0000	0.9883	1.0000
Peak 2	0.0050	0.4360	0.9998	0.2876	1.0000	1.0000	<0.0001
Peak 3	0.8719	<0.0001	0.1219	0.9976	1.0000	0.8319	<0.0001
Peak 4	0.0004	0.3473	0.9989	0.1291	1.0000	0.9644	<0.0001
Peak 5	0.0027	0.2890	0.9844	0.3088	1.0000	0.9986	<0.0001
Peak 6	<0.0001	0.4741	0.9463	0.2291	1.0000	0.6318	<0.0001
Peak 7	<0.0001	0.2647	0.8014	0.1616	1.0000	0.1271	<0.0001

## Data Availability

All data supporting the findings of this study are available from the corresponding authors upon request.

## References

[1] Zheng K., Bantog C., Bayer R. (2011). The impact of glycosylation on monoclonal antibody conformation and stability. MAbs.

[2] Boune S., Hu P., Epstein A.L., Khawli L.A. (2020). Principles of N-Linked Glycosylation Variations of IgG-Based Therapeutics: Pharmacokinetic and Functional Considerations. Antibodies.

[3] Zhou Q., Qiu H. (2019). The Mechanistic Impact of N-Glycosylation on Stability, Pharmacokinetics, and Immunogenicity of Therapeutic Proteins. J. Pharm. Sci..

[4] Zhang L., Luo S., Zhang B. (2016). Glycan analysis of therapeutic glycoproteins. MAbs.

[5] Reusch D., Haberger M., Maier B., Maier M., Kloseck R., Zimmermann B., Hook M., Szabo Z., Tep S., Wegstein J. (2015). Comparison of methods for the analysis of therapeutic immunoglobulin G Fc-glycosylation profiles—Part 1: Separation-based methods. MAbs.

[6] Ruhaak L.R., Zauner G., Huhn C., Bruggink C., Deelder A.M., Wuhrer M. (2010). Glycan labeling strategies and their use in identification and quantification. Anal. Bioanal. Chem..

[7] Anumula K.R., Dhume S.T. (1998). High resolution and high sensitivity methods for oligosaccharide mapping and characterization by normal phase high performance liquid chromatography following derivatization with highly fluorescent anthranilic acid. Glycobiology.

[8] (2024). ICH Q14: Analytical Procedure Development. https://www.ema.europa.eu/en/documents/scientific-guideline/ich-q14-guideline-analytical-procedure-development-step-5_en.pdf.

[9] Helali Y., Delporte C. (2024). Updates of the current strategies of labeling for N-glycan analysis. J. Chromatogr. B Anal. Technol. Biomed. Life Sci..

[10] New England Biolabs PNGase F. https://www.neb.com/en-us/products/p0705-pngase-f-glycerol-free.

[11] Agilent Endoglycosidases, Part Number: GKE-5006B. https://www.agilent.com/store/en_US/Prod-GKE-5006B/GKE-5006B.

[12] Promega PNGase F. https://www.promega.com/products/mass-spectrometry/glycosidases/pngase-f/?catNum=V4831.

[13] ThermoFisher Scientific Gibco™ PNGase F Glycan Cleavage Kit. https://www.thermofisher.com/order/catalog/product/A39245.

[14] N-Zyme Scientifics. PNGase F PRIME™ GLYCOSIDASE (Liquid Format). https://www.n-zymesci.com/glycosidases.

[15] Glycobiology Unit Conversion Chart/NEB. https://www.neb.com/en-us/tools-and-resources/usage-guidelines/glycobiology-unit-conversion-chart.

[16] Parr M.K., Schmidt A.H. (2018). Life cycle management of analytical methods. J. Pharm. Biomed. Anal..

[17] PNGase F Properties & Usage. https://www.neb.com/en-us/products/p0704-pngase-f#:~:text=Properties%20&%20Usage,are%20visualized%20by%20SDS%2DPAGE.

[18] Agilent Agilent AdvanceBio N-Glycanase (PNGase F, EDTA-Free), ≥2.5 U/mL. https://www.agilent.com/cs/library/datasheets/public/5994-1054EN.pdf.

[19] Badawy M.E.I., El-Nouby M.A.M., Kimani P.K., Lim L.W., Rabea E.I. (2022). A review of the modern principles and applications of solid-phase extraction techniques in chromatographic analysis. Anal. Sci..

[20] Tian Y., Zhou Y., Elliott S., Aebersold R., Zhang H. (2007). Solid-phase extraction of N-linked glycopeptides. Nat. Protoc..

[21] Dias N.C.P., Poole C.F. (2002). Mechanistic study of the sorption properties of OASIS^®^ HLB and its use in solid-phase extraction. Chromatographia.

[22] Tanna N., Plummer C. (2025). Oasis Prime HLB—The Fastest Way to Clean Samples. Waters Application Note. https://www.waters.com/content/dam/waters/en/app-notes/2025/720008684/720008684-en.pdf.

[23] ThermoFisher. https://www.thermofisher.com/order/catalog/product/60108-571.

[24] Young C., Condina M.R., Briggs M.T., Moh E.S.X., Kaur G., Oehler M.K., Hoffmann P. (2021). In-House Packed Porous Graphitic Carbon Columns for Liquid Chromatography-Mass Spectrometry Analysis of N-Glycans. Front. Chem..

[25] Yang X., Bartlett M.G. (2019). Glycan analysis for protein therapeutics. J. Chromatogr. B Anal. Technol. Biomed. Life Sci..

[26] Oh M.J., Seo Y., Kim U., An H.J. (2021). In-Depth Glycan Characterization of Therapeutic Glycoproteins by Stepwise PGC SPE and LC-MS/MS. Methods Mol. Biol..

[27] MilliporeSigma. https://www.sigmaaldrich.com/US/en/product/supelco/57088.

[28] Merck Merck Solid Phase Extraction Products. http://www.supelco.com.tw/C-02-SPE-Tube.pdf.

[29] Neville D.C., Coquard V., Priestman D.A., te Vruchte D.J., Sillence D.J., Dwek R.A., Platt F.M., Butters T.D. (2004). Analysis of fluorescently labeled glycosphingolipid-derived oligosaccharides following ceramide glycanase digestion and anthranilic acid labeling. Anal. Biochem..

[30] Zhang Q., Li H., Feng X., Liu B.F., Liu X. (2014). Purification of derivatized oligosaccharides by solid phase extraction for glycomic analysis. PLoS ONE.

[31] Lauber M.A., Koza S.M., Fountain K.J. (2013). Single-Use and High-Throughput HILIC SPE Device Formats and an IgG Control Standard for Facilitating N-Glycan Analyses. https://www.waters.com/content/dam/waters/en/app-notes/2013/720004716/720004716-en.pdf.

[32] Yehuda S., Padler-Karavani V. (2020). Glycosylated Biotherapeutics: Immunological Effects of N-Glycolylneuraminic Acid. Front. Immunol..

[33] Luo S., Zhang B. (2024). Benchmark Glycan Profile of Therapeutic Monoclonal Antibodies Produced by Mammalian Cell Expression Systems. Pharm. Res..

[34] Ghaderi D., Taylor R.E., Padler-Karavani V., Diaz S., Varki A. (2010). Implications of the presence of N-glycolylneuraminic acid in recombinant therapeutic glycoproteins. Nat. Biotechnol..

[35] Gimenez E., Sanz-Nebot V., Rizzi A. (2013). Relative quantitation of glycosylation variants by stable isotope labeling of enzymatically released N-glycans using [12C]/[13C] aniline and ZIC-HILIC-ESI-TOF-MS. Anal. Bioanal. Chem..

[36] Prasher P., Sharma M. (2021). Medicinal chemistry of anthranilic acid derivatives: A mini review. Drug Dev. Res..

[37] Eberle M., Wasylenko J.T., Kostelac D., Kiehna S., Schellinger A., Zhang Z., Ehrick J.D. (2025). A Modern Framework for Analytical Procedure Development and Lifecycle Management Based on ICH Q14 Principles. Anal. Chem..

